# Special Issue: “Electric, Magnetic, and Electromagnetic Fields in Biology and Medicine: From Mechanisms to Biomedical Applications”

**DOI:** 10.3390/ijerph16224548

**Published:** 2019-11-18

**Authors:** Maria Rosaria Scarfì, Mats-Olof Mattsson, Myrtill Simkó, Olga Zeni

**Affiliations:** 1 Institute for Electromagnetic Sensing of the Environment, National Research Council, Via Diocleziano, 328-80124 Naples, Italy; zeni.o@irea.cnr.it; 2 SciProof International AB, Vaktpoststigen 4, 83132 Östersund, Sweden; mats-olof.mattsson@sciproof-international.se (M.-O.M.); myrtillsimko@gmail.com (M.S.)

**Keywords:** electromagnetic bio-effects, interaction mechanisms, environmental health, electromagnetic field modelling, exposure assessment, diagnostic and therapeutic applications, experimental studies

## 1. Special Issue Rationale

The last decades have seen a huge increase in applications and devices using and emitting non-ionizing radiation, otherwise referred to as “electromagnetic fields” (EMF). This includes, e.g., the distribution and use of electricity and information and communication technologies. Present and foreseeable future technologies employ different parts of the electromagnetic (EM) spectrum, from static electric and magnetic fields, via low frequency fields to high frequency EMF encompassing millimeter waves and THz fields. Thus, humans are exposed to various electric, magnetic, and EMF in the course of everyday life. In addition, these kinds of non-ionizing radiation are also successfully employed in biomedical applications, for both diagnostic and therapeutic purposes, causing exposure in specific occupational environments. As a consequence, public and scientific concern with respect to the possible adverse health effects of EMF occurring after exposure to levels below those inducing known acute effects has been rising. A large body of literature dealing with experimental as well as clinical and population-based investigations into the biological and health effects of such fields has yielded inconsistent and often conflicting information. Moreover a comprehensive understanding of the mechanisms of interaction remains to be elucidated. Therefore, there is great interest in evaluating the induced biological responses from the point of view of the associated interaction mechanisms. 

## 2. About the Papers of This Special Issue

The call for articles on “Electric, Magnetic, and Electromagnetic Fields in Biology and Medicine: From Mechanisms to Biomedical Applications” resulted in a total of 14 accepted manuscripts: 10 regular papers, one communication and three review papers. On the whole, the following issues were addressed:

(1) EMF Exposure Assessment

In the characterization of human exposure to EMF, children’s exposure to extremely low frequency (ELF) magnetic fields has been a matter of debate since several meta-analyses reported on an increased risk of childhood leukemia’s for daily average exposure above 0.4 µT without any causal relationship. This issue was covered in two papers by the same research group. Bonato et al. [1] propose a new method for the characterization of children’s exposure, which relies on personal measurements and employs a stochastic approach based on segmentation that resulted in the identification of the parameters most affecting the level of children’s exposure. Tognola et al. [2], acharacterized a real-life ELF exposure scenario by applying a machine learning approach on personal exposure measurements from 977 children in France. The impact of only outdoor sources of exposure was considered.

The measurements of personal exposure to radiofrequency (RF) EMF is a challenging task in epidemiological studies and was covered in two papers and one review article. Zeleke et al. [3] recorded and analyzed measurements of personal RF-EMF exposure levels from a wide range of frequency bands from 63 participants over a range of 27.4 ± 4.5 h. Liu et al. [4] investigated the applicability of an efficient whole-body individual modelling method for the assessment of RF exposure in the case of patient exposure under MRI examination. Chiaramello et al. [5] presented an overview of the papers published in the last ten years and address RF-EMF exposure assessment from different sources in indoor environments.

(2) Biological Effects of EMF Exposure and Health Risk Evaluation

An in vitro study by Sannino et al. [6] indicated a possible involvement of DNA repair mechanisms in the RF-induced adaptive response, a phenomenon by which pre-exposure of different cell cultures to RF fields under specific conditions is capable of reducing the damage induced by subsequent treatment with genotoxic agents.

Three studies report on the results of in vivo investigations carried out on laboratory animals. In particular, Guo et al. [7] investigated the effects of one-month exposure to a 220 MHz pulsed modulated RF field at the power density of 50 W/m^2^ on the sperm quality in male adult rats, which was found to be impaired by the exposure. These exposure conditions were chosen by the authors since they are realistic under certain occupational conditions in which workers are exposed to high intensity RF fields.

The Medaka fish was chosen in the paper by Sun et al. [8] to address the effect of long term (entire development period) static magnetic field exposure at the mT level on embryo development and behavioral response. Development was not affected whereas swimming velocity was reduced in the exposed group.

Sienkiewicz and van Rongen [9] reviewed the evidence on the effects of exposure to RF fields, mostly associated with mobile phone technology, on the cognitive behavior of laboratory animals. Vornoli et al. [10] reviewed the currently available evidence from in vivo studies on carcinogenicity and reproductive toxicity studies in order to summarize the contribution of experimental research to the prevention of the adverse effects of RF radiation on human health.

Two studies were carried out on human volunteers. In Loughran et al. [11], the influence of RF-EMF on electroencephalogram (EEG) readings was investigated in 36 healthy adults participating in a randomized, double-blind provocation study with the aim to find out if a thermal mechanism is involved in nervous system responses to RF-EMF. Indeed, the results suggest such an involvement.

Vecsei et al. [12] studied the effects of acute exposure from long term evolution (LTE) mobile phone EMF on the thermal pain threshold in healthy young adults and no effects of exposure were seen.

A couple of other relevant papers are collected in this special issue that address the topic of the extrapolation of the outcome of animal studies to humans [13], and the identification and description of methods using non-ionizing radiation (NIR) such as EMF and optical radiation in Swedish health care [14]. Specifically, Kodera and Hirata computationally estimated the thermal time constants of temperature elevation in human head and rat models exposed to dipole antennas at 3–10 GHz [13], while Hansson Mild and coworkers identified three applications in Swedish health care (magnetic resonance imaging (MRI), transcranial magnetic stimulation (TMS), and electrosurgery) where acute effects at existing exposure levels could not be ruled out [14]. 
